# Hemostatic efficacy of a flowable collagen-thrombin matrix during coronary artery bypass grafting: a double-blind randomized controlled trial

**DOI:** 10.1186/s13019-023-02196-3

**Published:** 2023-06-15

**Authors:** Hyo-Hyun Kim, Kang Ju Lee, Dae Ryong Kang, Jun Hyeok Lee, Young-Nam Youn

**Affiliations:** 1 Division of Cardiovascular Surgery, Severance Cardiovascular Hospital, Yonsei University College of Medicine, Yonsei University Health System, 250 Seongsanno, Seodaemun-gu, Seoul, 03722 Republic of Korea; 2 Department of Biostatistics, Wonju College of Medicine, Yonsei University, Wounju, Republic of Korea; 3 Department of Cardiothoracic Surgery, Ilsan Hospital, National Health Insurance Service, Goyang-si, 10444 Republic of Korea

**Keywords:** Coronary artery bypass, Off-pump, Hemostasis, Hemorrhage, Bleeding time, Blood transfusion

## Abstract

**Background:**

Flowable hemostatic agents have the advantage of being able to be applied to irregular wound surfaces and difficult to reach areas. We sought to compare the effectiveness and safety of the flowable hemostatic sealants Collastat® (collagen hemostatic matrix, [CHM]) and Floseal® (gelatin hemostatic matrix, [GHM]) during off-pump coronary artery bypass (OPCAB).

**Methods:**

In this prospective, double-blind, randomized controlled trial, 160 patients undergoing elective OPCAB surgery were enrolled between March 2018 and February 2020. After primary suture of the aortocoronary anastomosis, an area of hemorrhage was identified, and patients received either CHM or GHM (n = 80, each). Study endpoints were the following: proportion of successful intraoperative hemostasis and time required for hemostasis overall postoperative bleeding, proportion of transfusion of blood products, and surgical revision for bleeding.

**Results:**

Of the total patients, 23% were female, and the mean age was 63 years (range 42–81 years). Successful hemostasis proportion within 5 min was achieved for 78 patients (97.5%) in the GHM group, compared to 80 patients (100%) in the CHM group (non-inferiority *p* = 0.006). Two patients receiving GHM required surgical revision to achieve hemostasis. There were no differences in the mean time required to obtain hemostasis [GHM vs. CHM, mean 1.49 (SD 0.94) vs. 1.35 (0.60) min, *p* = 0.272], as confirmed by time-to-event analysis (*p* = 0.605). The two groups had similar amounts of mediastinal drainage for 24 h postoperatively [538.5 (229.1) vs. 494.7 (190.0) ml, *p* = 0.298]. The CHM group required less packed red blood cells, fresh frozen plasma, and platelets for transfusion than the GHM group (0.5 vs. 0.7 units per patient, *p* = 0.047; 17.5% vs. 25.0%, *p* = 0.034; 7.5% vs. 15.0%, *p* = 0.032; respectively).

**Conclusions:**

CHM was associated with a lower need for FFP and platelet transfusions. Thus, CHM is a safe and effective alternative to GHM.

*Trial registration*: ClinicalTrials.gov, NCT 04310150.

## Background

Hemostasis, a key surgical procedure, is even more crucial in cardiac surgery. High-pressure anastomoses and suture lines within the cardiac chambers or the great vessels are created during virtually all cardiac surgical procedures [1]. Failure to achieve adequate hemostasis during surgery increases the complications from excessive bleeding, transfusion of blood products, and intensive care unit stays and the risk of mortality. [2] Fast intraoperative hemostasis reduces both the amount of blood lost and the need for perioperative blood transfusions. Furthermore, the evolution of many surgical procedures to using smaller, more minimally invasive incisions or approaches creates potentially high-risk settings since the ability to access and control persistent bleeding sites is diminished. The availability and development of reliable products to control bleeding in this setting will potentially enhance the safety of these procedures. [3]

Patients undergoing cardiac surgery with or without cardiopulmonary bypass are at risk for excessive bleeding and the associated complications. Allogeneic blood transfusion is associated with immunomodulation and infection. Moreover, the cost incurred per hospitalization event for bleeding complications or transfusions in cardiac cases is reported to be 10,000 USD. [4]

Over the years, several topical hemostatic agents have been developed to control troublesome intraoperative bleeding [5]. The commercial topical hemostatic agent, Floseal® (Baxter International, Inc., Deerfield, IL, USA), a gelatin hemostatic matrix (GHM), is a combination of bovine-derived gelatin and pharmacologically active bovine thrombin [6, 7]. Because in vivo hemostatic agents are more likely to remain in the body after treatment, it is necessary to reduce any side effects by using highly biocompatible materials. Recently, a hemostatic agent that combines porcine-derived collagen with bovine thrombin into a collagen hemostatic matrix (CHM) with a low antigenicity has been developed (CollaStat, Dalim Tissen Co., Ltd., Seoul, Korea) [8].

Coagulopathy resulting in excessive bleeding or an increased need for blood transfusion during vascular and cardiac surgery is common [9]. Fibrin and thrombin sealants are used topically to reduce bleeding. However, there are concerns about their effectiveness and about adverse effects, including viral activity, and the antigenicity of bovine thrombin or aprotinin used in the majority of commercially available fibrin sealants [10, 11]. Thus, it should be established whether fibrin and thrombin sealants are safe and effective.

During off-pump coronary artery bypass (OPCAB), serious trauma (sternotomy, internal mammary artery, saphenous vein or radial artery graft harvesting, pericardiotomy, and heart manipulation) and heparin and protamine exposure activate coagulation by releasing tissue factors and activating extrinsic pathways [12]. Therefore, blood transfusions are still needed for OPCAB, and complications after blood infusion have become one of the main concerns with OPCAB [13]. A greater activation level of fibrinogen and other acute-phase proteins has been observed in OPCAB compared with on-pump CABG, which may lead to higher thromboembolic event risk in OPCAB [14]. Therefore, the efficacy and safety profiles of hemostat in OPCAB surgery.

The key factors in a surgeon’s selection of an appropriate topical hemostatic agent include the procedure type, their product experience and personal preference, the product’s cost, and the severity of the bleeding. This study aimed to compare the effectiveness and safety of the topical hemostatic agents CHM (experimental group) and GHM (control group) in patients who underwent OPCAB. We intended to investigate the hemostatic efficacy of a collagen-based hemostat during coronary artery bypass surgery (CABG) by comparing it with that of a conventional, flowable hemostat.

## Methods

### Patients

A total of 160 patients were enrolled over a 24-month period in a prospective, double-blind, single-center randomized controlled trial between March 2018 and February 2020. The Institutional Review Board (IRB) at our site approved the study prior to patient enrollment (Severance Hospital, South Korea, IRB number; 1-2017-0094). The study was performed in accordance with the ethical standards laid down in the 1964 Declaration of Helsinki and its later amendments. This study met the criteria of a primary registry of the WHO (ClinicalTrials.gov, NCT 04310150) before patient recruitment. Informed consent was obtained prior to the operative procedure. After explaining the randomized study of hemostatic agents, along with the procedure description, we obtained the patient’s consent. The inclusion criteria for enrollment eligibility specified patients aged 19 years or older who underwent elective OPCAB surgery for multivessel coronary artery disease. Patients were excluded if they were pregnant or had a known sensitivity to any components of the bovine thrombin preparations or to the porcine or bovine materials. Patients who were taking antithrombotic or antiplatelet agents for more than one week, except for aspirin, or who had a hematologic disease were also excluded from this study.

### Procedure and assessment of hemostasis

Baseline testing within 24 h prior to surgery included a complete blood count with differential, the activated partial thromboplastin time (aPTT), the prothrombin time, an electrolyte panel and a hepatic or renal panel. Patient enrollment occurred in the operating room when an aortocoronary bypass was determined. After full median sternotomy, heparin (0.7–1.0 mg/kg) was administered to achieve the target activated clotting time (ACT; > 300 s).

In all patients, a saphenous vein or radial artery graft was anastomosed to the aorta using the Heartstring device (MAQUET Holding B.V. & Co. KG, Rastatt, Germany) at the beginning of a surgery. As soon as identification of bleeding of the aorto-graft anastomosis site, we applied treatment regimen with one of the two hemostatic agents, GHM or CHM, using a block randomization system. The topical hemostatic agents were specially prepared with the same dose and same color. The containers containing the solutions were then sent to the operating room with only an ID number, which marking was removed, making it impossible to identify them when used in the operating room. These hemostatic agents were delivered to the operating room in a sealed envelope by the nurse. The selected agent was delivered to the site of bleeding, followed by light compression with a wet gauze until hemostasis was achieved.

**Fig. 1:**
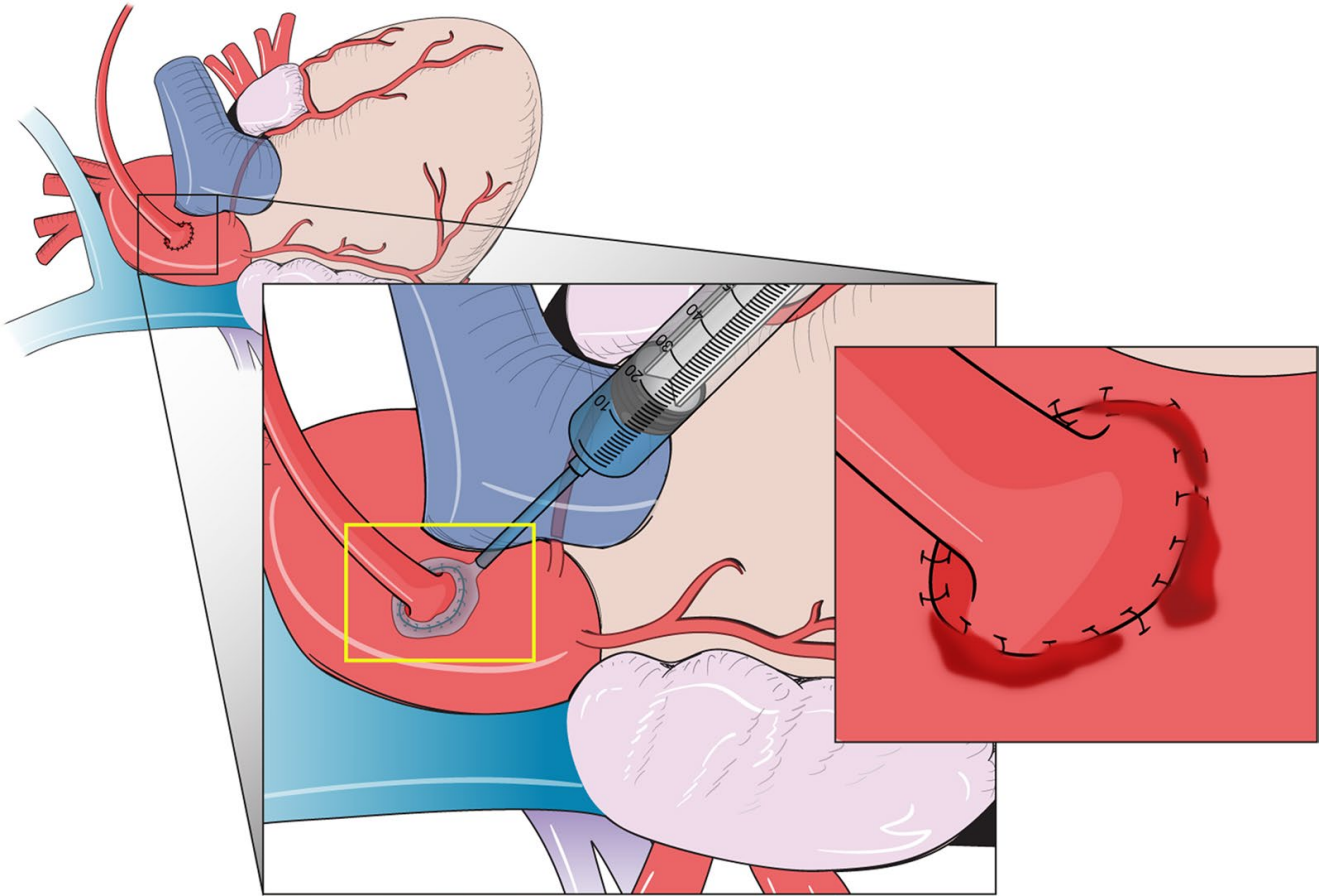
Application of CHM to an aortocoronary anastomosis. The yellow box depicts the application process. The inset shows the treatment area

We defined the hemostatic status on aorto-graft anastomosis site using the three levels (0: Dry, 1: Oozing, and 2: Pooling) of the Surface Bleeding Severity Scale. [15] The hemostatic agent, CHM or GHM, was applied with pressure to the target area for 1 min (Fig. 1). If hemostasis was achieved within this time, the time to hemostasis was recorded as 1 min. If hemostasis was not achieved, the treatment was re-applied every minute up to 5 min. If hemostasis was not achieved within 5 min, surgical revision was performed using conventional 6–0 polypropylene sutures (Prolene; Ethicon, Inc, Somerville, NJ).

Protamine was administered at the end of the OPCAB procedure. After the completion of the anastomoses, residual heparin was reversed with 1 mg of protamine for every mg of heparin used for systemic heparinization. Following protamine administration, the ACT was assessed at 3, 15, and 30 min. If an additional dose of protamine was required, the quantity of protamine and resultant ACT 10 min after completion of the additional dose were recorded. The pericardium was loosely closed after the surgery, the midline sternotomy was closed in layers, and two mediastinal drains were retained.

Participants in both groups took 100 mg aspirin and 75 mg clopidogrel daily from the first postoperative day. Patients with hemoglobin (Hb) values below 60 g/L received transfusion therapy. In stable patients with Hb values between 60 and 100 g/L, an evaluation of the patients’ clinical status was necessary to determine if transfusion was warranted. Transfusion of fresh frozen plasma (FFP) was indicated for the following: an international normalized ration (INR) greater than 1.5; microvascular bleeding in patients who underwent massive transfusion; and acute disseminated intravascular coagulation in the presence of ongoing bleeding. Transfusion of platelet concentrates was indicated if the platelet count was below 50 × 10^9^/L and there was active bleeding. [1, 6, 17]

### Drain management

Two round 32F silicone thoracic catheters were placed in the retrosternal space in all patients. The mediastinal drains were connected to a disposable dry suction control chamber (OASIS Dry Suction Water Seal Chest Drain; Maquet, Rastatt, Germany) with 20 cmH_2_O of suction. The drains were retained for at least 24 h postoperatively and removed when there was < 150 cc of daily drainage with a trend of decreasing effusion.

### Definition of successful hemostasis and endpoints

For the clinical application of the hemostatic agents, successful hemostasis was achieved when there was cessation of visible bleeding after completion of the hemostatic agent administration.

The primary end points were the proportion of patients with complete hemostasis within 5 min for the aortocoronary anastomosis sites treated with GHM or CHM and the proportion of patients with complete hemostasis evaluated at 1, 2, and every minute up to 5 min. The secondary end points were the time required for hemostasis, the amount of blood loss on the operative day, the amount of blood products transfused both intraoperatively and postoperatively, the surgical revision rate for bleeding, the total length of intensive care unit (ICU) stay, and the rate of postoperative morbidity/ mortality. All patients were followed during their hospital stay by the same member of the surgical team, who filled out a protocol for pre- and postoperative data comparison.

### Statistical analysis

The sample size for the study was calculated based on a level 0.025 test to exclude a probability of hemostasis within 5 min that was 10% less among subjects treated with CHM compared to those treated with GHM. The 10% non-inferiority margin is based on an FDA guidance for industry on non-inferiority clinical trials [18]. In historic institutional data, the sample size was based on a power calculation that assumed an equivalent performance of the experimental and control groups at 88%. Using the sample size formula, the sample size was calculated to be 80 patients in each group.

Patients were randomized 1:1 using computer-generated permuted block randomization. Lists with a block size of 4 were generated at the initiation of the study using the RandList software (Datlnf GmbH, Tübingen, Germany). The results for the effective achievement of the primary end point were statistically assessed using an intention-to-treat analysis. The time to cessation of bleeding was compared using the Gehan-Wilcoxon test. The between-group difference of hemostatic rate and corresponding 95% confidence intervals (CIs) for this difference were calculated. CHM was considered non-inferior to GHM if one-sided P value was < 0.025. because the non-inferiority test is one-sided by nature, a clinical significance level of 0.025 was used for the non-inferiority test to keep the duality between the test and 95% CI as well as the same level of rigor of the two-sided test. The baseline and short-term clinical follow-up data were compared between the groups using the Fisher’s exact test. The number of distal anastomoses was analyzed by nonparametric analysis of variance (Kruskal–Wallis ANOVA). The IBM SPSS Statistics package (version 23.0, IBM-SPSS Inc., Armonk, NY) and SAS System software (version 9.3, SAS Institute, Cary, NC) were used for all statistical analyses.

## Results

**Fig. 2:**
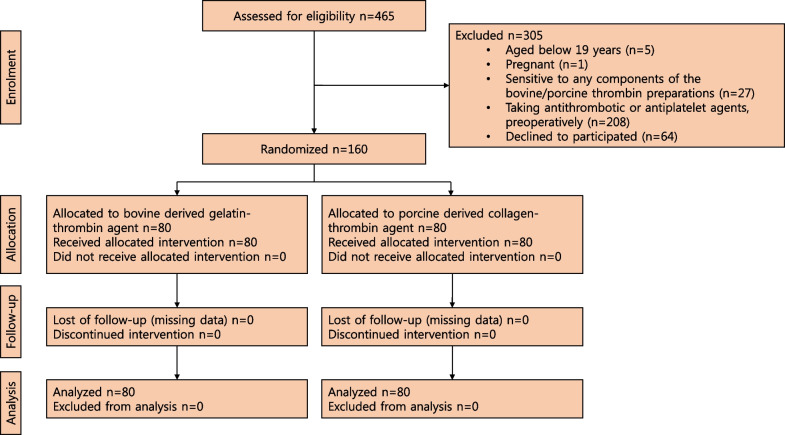
Study flow diagram. CAOD: coronary arterial obstructive disease, OPCAB: Off-pump coronary artery bypass

The study design is summarized in Fig. 2. A total of 465 patients were screened for eligibility. Of these, 241 patients were excluded because they failed to meet the inclusion criteria. Of the remaining 224 patients, 64 were not enrolled because they refused consent. A total of 160 patients were enrolled; 80 were randomized to the GHM group and 80 to the CHM group.

The two study groups were comparable with respect to baseline characteristics, as outlined in Table 1. The mean age of the patients was 63.4 ± 7.2 years, and 123/160 (76.9%) were men. All patients had a history of taking aspirin. The preoperative hemoglobin and platelet counts, the type of anastomotic grafts, and the total number of distal anastomoses were not significantly different between the groups. The intraoperative data, including the peak ACT and hemostatic values, were also similar between the two groups (Table 2).

**Table 1:** Patient demographics and preoperative medications

Variable	GHM N (%), mean (SD), or median [Q1, Q3]	CHMN (%), mean (SD), or median [Q1, Q3]	P value
*Demographic data (N = 80, respectively)*			
Age (mean ± SD)	63.62 ± 7.1	63.1 ± 7.3	0.649
Sex			
Men (n, %)	62 (77.5)	61 (76.3)	0.500
Women (n, %)	18 (22.5)	19 (23.7)	
BMI (kg/m^2^, mean ± SD)	24.5 ± 3.5	24.6 ± 3.1	0.838
Hypertension (n, %)	42 (52.5)	48 (60.0)	0.505
Diabetes mellitus (n, %)	38 (47.5)	44 (55.0)	0.508
Chronic kidney disease (n, %)	14 (17.5)	6 (7.5)	0.194
BUN (mg/dL, mean ± SD)	20.1 ± 11.8	17.2 ± 7.4	0.189
Creatinine (mg/dL, mean ± SD)	1.6 ± 2.2	1.5 ± 1.0	0.113
Hyperlipidemia (n, %)	8 (10.0)	12 (15.0)	0.519
Previous AMI (n, %)	2 (2.5)	4 (5.0)	0.241
PCI history (n, %)	16 (20.0)	18 (22.5)	0.790
*Acute coronary syndrome*			
Unstable angina (n, %)	26 (32.5)	24 (30.0)	0.999
STEMI (n, %)	2 (2.5)	10 (12.5)	0.201
NSTEMI (n, %)	20 (25.0)	16 (20.0)	0.790
LVEF (%, mean ± SD)	56.2 ± 14.0	53.1 ± 13.7	0.757
LM disease (n, %)	16 (20.0)	22 (27.5)	0.708
Hemoglobin (g/dL, mean ± SD)	12.4 ± 2.2	12.4 ± 2.4	0.507
Hematocrit (%, mean ± SD)	36.3 ± 6.7	36.8 ± 7.0	0.436
Platelet (10^–3^/μL, mean ± SD)	251 [101, 340]	282 [113, 376]	0.480
Prothrombin time (INR, mean ± SD)	1.0 ± 0.1	1.0 ± 0.1	0.270
aPTT (mean ± SD)	49.8 ± 21.3	44.8 ± 17.4	0.094
Creatinine (mg/dL, mean ± SD)	1.0 ± 0.5	1.1 ± 1.4	0.162
*Preoperative medications*			
Aspirin (n, %)	80 (100)	80 (100)	-
Heparin, intravenous (n, %)	46 (57.5)	36 (45.0)	0.371
β-blocker (n, %)	20 (25.0)	22 (27.5)	0.525
ACEI/ARB (n, %)	6 (7.5)	8 (10.0)	0.226
Calcium channel blocker (n, %)	16 (20.0)	16 (15.0)	0.428
Statins (n, %)	40 (50.0)	32 (40.0)	0.311
Nitrates (n, %)	2 (2.5)	4 (5.0)	0.573

*ACEI angiotensin-converting enzyme inhibitor, *AMI* acute myocardial infarction, *aPTT* activated partial thromboplastin time, *ARB* angiotensin II receptor blocker, *BMI* body mass index, *BUN* blood urea nitrogen, *CHM* collagen hemostatic matrix, *GHM* gelatin hemostatic matrix, *INR* international normalized ratio, *LM* left main, *LVEF* left ventricular ejection fraction, *NSTEMI* non ST segment elevation myocardial infarction, *PCI* percutaneous coronary intervention, *STEMI* ST elevation myocardial infarction

**Table 2:** Intraoperative data

Variable	GHM N (%), mean (SD), or median [Q1, Q3]	CHM N (%), mean (SD), or median [Q1, Q3]	*P* value
*Surgical data*			
Graft of aortocoronary anastomosis (n, %)			
SVG	72 (90.0)	73 (91.2)	0.500
RA	8 (10.0)	7 (8.8)	
No. of distal anastomoses (mean ± SD)	3.3 ± 0.8	3.4 ± 0.8	0.632
Total operative time (min, mean ± SD)	248.9 ± 33.3	253 ± 43.0	0.583
*Intraoperative data (mean* ± *SD)*			
Body temperature, °C	36.8 ± 0.3	36.7 ± 0.3	0.094
Intraoperative bleeding (ml, mean ± SD)	395 (120, 645)	350 (140, 615)	0.704
Heparin loading (units, mean ± SD)	5655 (4500, 7500)	5535 (4400, 7300)	0.409
Peak ACT during surgery (mean ± SD)	279.4 ± 40.2	281.1 ± 44.6	0.540
Last ACT during surgery (mean ± SD)	139.5 ± 10.9	141.6 ± 10.3	0.673
Protamine for reversal of heparin (mg, mean ± SD)	23.3 ± 3.5	22.1 ± 5.6	0.068
Successful hemostasis (n, %)	78 (97.5)	80 (100)	0.497
Revision suture for bleeding (n, %)	2 (2.5)	0 (0)	0.497

*ACT activated clotting time, *CHM* collagen hemostatic matrix, *GHM* gelatin hemostatic matrix, *RA* radial artery, *SVG* saphenous vein, body temperature was measured in bladder temperature just before aorta anastomosis

The baseline bleeding characteristics, expressed as oozing or pooling, were similar between the two groups [64 (80.0%) vs. 16 (20.0%) in the GHM group and 60 (75.0%) vs. 20 (25.0%) in CHM, respectively). The rate of successful hemostasis within 5 min was 97.5% (78/80) in the GHM group vs. 100% (80/80) in the CHM group (*p* = 0.497). For two patients who received GHM, hemostasis was achieved with suture revision at 2 and 3 min from topical application, respectively. Since the anastomosis site was under high pressure, especially in a porcelain or severely calcified aorta, hemostasis was needed to prevent a massive pulsatile hemorrhage and revision was performed according to the surgeon’s judgment.

**Fig. 3:**
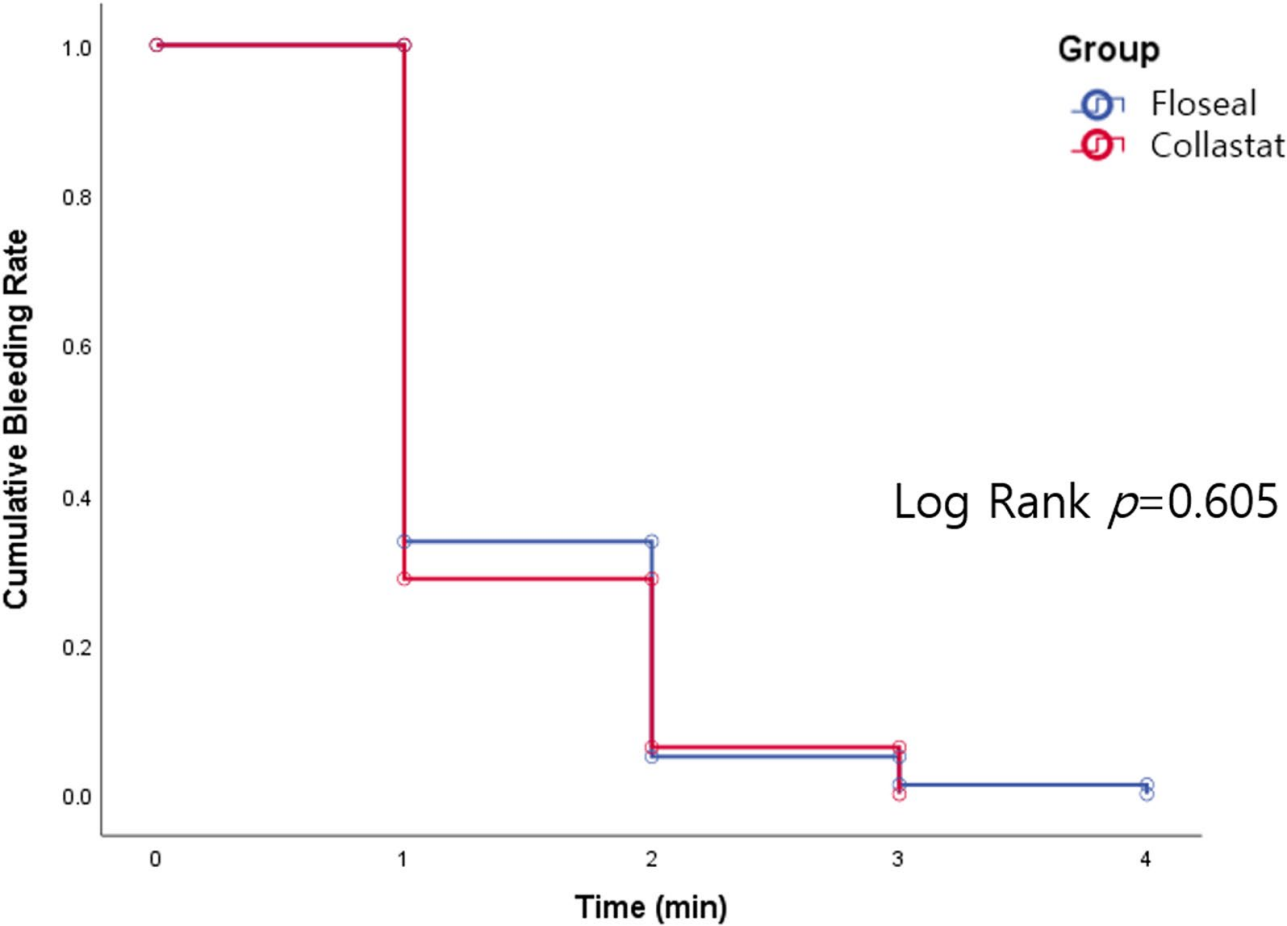
Kaplan–Meier plot for the time to achieve successful hemostasis for the aortocoronary anastomosis site treated with CHM (experimental group) or GHM (control group)

CHM demonstrated non-inferiority at 5 min compared to GHM (*p* = 0.006 for non-inferiority) (Table 3). Notably, the mean time required to obtain hemostasis was similar between the two groups (GHM vs. CHM, mean 1.49 (SD 0.94) vs. 1.3 (0.60) min, *p* = 0.272), which was confirmed by the time-to-event analysis (Fig. 3, *p* = 0.605).

**Table 3:** Comparison (non-inferiority) of GHM to CHM for success at achieving hemostasis within 5 min

Primary endpoint	GHM (n = 80)	CHM (n = 80)	Difference (95% CI)	*P* value
*Baseline bleeding characteristics*				
Oozing	64 (80.0)	60 (75.0)	5.0%	0.303
Pooling	16 (20.0)	20 (25.0)	5.0%	0.303
Time to successful hemostasis (min, mean ± SD)	1.49 ± 0.94	1.35 ± 0.60	0.13 ± 0.19	0.272
1 min (n, %)	53/80 (66.3)	57/80 (71.3)	5.0%	–
2 min (n, %)	75/80 (93.8)	75/80 (93.8)	0.0%	–
3 min (n, %)	77/80 (96.3)	80/80 (100)	3.8%	–
4 min (n, %)	78/80 (97.5)	80/80 (100)	2.5%	–
5 min (n, %)	78/80 (97.5)	80/80 (100)	2.5% (-3.8, 11.7)	0.006

*CHM collagen hemostatic matrix, *CI* confidence interval, *GHM* gelatin hemostatic matrix

In the CHM group, one patient died due to sepsis worsened by *Pseudomonas aeruginosa* pneumonia, 26 d postoperatively. Since this patient did not receive a blood transfusion during the immediate postoperative period and had no allergic drug reactions, the outcome was determined to be unrelated to an adverse reaction to CHM. One patient in the GHM group experienced cardiogenic shock caused by ventricular arrhythmia. Another patient in the GHM group was diagnosed with postoperative myocardial infarction but improved after medical treatment and did not require repeat revascularization (Table 4).

**Table 4:** Adverse events

Variable	GHM (n = 80)	CHM (n = 80)	P value
AE	48 (60)	48 (60)	0.369
ADE	0	0	–
SAE	0	1 (1.25)	0.999
30-day mortality	0	1 (1.25)	0.999
Cardiogenic shock	1 (1.25)	0	0.999
Postoperative myocardial infarction	1 (1.25)	0	0.999
Repeated revascularization	0	0	–
Fever	16 (20.0)	10 (12.5)	0.237
Infection, reported by culture study	8 (10.0)	14 (17.5)	0.225
Sepsis	0	1 (1.25)	0.999
Pneumonia	4 (5.0)	6 (7.5)	0.359
Wound, sternum	0	0	–
Wound, leg	2 (2.5)	6 (7.5)	0.179
Urinary tract	2 (2.5)	2 (2.5)	0.999
Pleural effusion requiring chest tube drainage	6 (7.5)	2 (2.5)	0.179
Pericardial effusion requiring pericardiocentesis	0	2 (2.5)	0.999
New onset arrhythmia	4 (5.0)	0	0.494
Stroke	0	0	–
Seizure	0	2 (2.5)	0.999
Gastrointestinal tract bleeding	0	2 (2.5)	0.999
Nausea	24 (30.0)	22 (27.5)	0.999
Vomiting	2 (2.5)	2 (2.5)	0.999

*ADE adverse device effect, *AE* adverse events, *CHM* collagen hemostatic matrix, *fever*: any body temperature above 38 °C, *GHM* gelatin hemostatic matrix, *SAE* serious adverse event

The amount of mediastinal drainage in the 24 h after surgery was not significantly different between the two groups [GHM vs. CHM, mean 538.5 (SD 229.1) vs. 494.7 (190.0) ml, *p* = 0.298; Table 5]. However, there was a significant difference in the average number of packed red blood cell (RBC) units transfused per patient between the two groups (GHM vs. CHM, 0.7 vs. 0.5 units, *p* = 0.047). Furthermore, FFP and platelets were transfused less frequently in the CHM group than in the GHM group [14 (17.5%) vs. 20 (25.0%), *p* = 0.034 and 6 (7.5%) vs. 12 (15.0%), *p* = 0.032; respectively). No life-threatening bleeding was noted during the study period, and the occurrence of minor bleeding (mediastinal drainage) was not significantly different between the GHM and CHM groups [n = 1 (1.25%) vs. 2 (2.5%), *p* = 0.897, respectively]. For both groups, there were no anaphylactic or severe systemic reactions to human blood products and the lengths of the ICU and the hospital stays were similar.

**Table 5:** Early outcomes

Variable	GHM N (%), mean (SD), or median [Q1, Q3]	CHM N (%), mean (SD), or median [Q1, Q3]	*P* value
Mediastinal drains 24 h postoperatively (ml) (mean ± SD)	538.5 ± 229.1	494.7 ± 190.0	0.298
Blood transfusion rates (n, %)	18 (22.5)	14 (17.5)	0.143
Packed RBC (n, %)	5 (6.3)	3 (3.8)	0.067
Packed RBC, ml (mean ± SD)	220 (200, 400)	150 (100, 320)	0.047
FFP (n, %)	20 (25.0)	14 (17.5)	0.034
FFP, unit (mean ± SD)	2.8 ± 2.4	2.1 ± 3.7	0.781
Platelets (n, %)	12 (15.0)	6 (7.5)	0.032
Platelets, unit (mean ± SD)	8.5 ± 5.7	7.8 ± 6.1	0.672
ICU stay, day (mean ± SD)	2.8 ± 1.4	2.3 ± 1.6	0.093
Hospital stay, day (mean ± SD)	8.2 ± 13.8	7.5 ± 10.3	0.520
^a^Major complications (n, %)	4 (5.0)	2 (2.5)	0.323
^b^Minor complications (n, %)	6 (7.5)	14 (17.5)	0.176
In hospital mortality	0	2 (2.5)	0.314

*CHM collagen hemostatic matrix, *FFP* fresh frozen plasma, *GHM* gelatin hemostatic matrix, *ICU* intensive care unit, *RBC* red blood cell

^a^Major postoperative complications: myocardial infarction, sepsis, shock, stroke

^b^Minor postoperative complications: inotropic support lasting more than 24 h, renal failure, respiratory insufficiency

## Discussion

This study was designed as a prospective, randomized study to compare CHM with a commonly used hemostatic agent, GHM, during CABG. Active flowable hemostatic matrices (CHM or GHM) contain thrombin and a particulate carrier in a single application product. These products work by blocking blood flow and actively converting blood fibrinogen into fibrin at the site of bleeding [16, 17]. In our study, the two groups receiving active, flowable hemostatic agents were compared to assess the hemostatic effect, and the results were similar between the groups. Furthermore, the CHM group demonstrated a reduction in the rate of required RBC, FFP and platelet transfusions. These findings suggest a potential efficacy of CHM for achieving hemostasis during CABG.

Several prospective, randomized, controlled trials across numerous surgical areas have reported an active, flowable hemostatic matrix (GHM) to be a more effective hemostat, demonstrating faster hemostasis and better outcomes, than passive, non-flowable hemostatic agents (e.g., Gelfoam® an absorbable gelatin sponge, The Upjohn Co. Kalamazoo, MI, USA; Surgicel®, an oxidized regenerated cellulose, Jonson & Johnson Products, Inc, New Brunswick, NJ, USA) [21, 22]. Some of these studies also indicate that the use of an active, flowable hemostatic matrix is associated with fewer post-surgical complications and shorter surgical times compared to other common, passive, topical hemostats [23, 24]. In our study, both CHM and GHM showed complete cessation of bleeding within 4 min of application at aortocoronary anastomoses sites and few post-surgical complications.

Few studies have compared the clinical performance and outcomes of active, flowable, topical hemostatic matrices in cardiac surgery [22, 23]. Two studies compared GHM and Surgiflo® (thrombin-gelatin hemostatic matrix; Ethicon, Somerville, USA) in a porcine model and reported that GHM stopped bleeding more effectively than Surgiflo®. [27, 28] Scott et al. analyzed why GHM was associated with fewer negative outcomes than Surgiflo® [23]. These revealed that the performance differences of these active, flowable hemostats may be due to the composition of the gelatin granules. However, in our study, gelatin granule characteristics, such as a porcine (CHM) versus a bovine (GHM) source, did not contribute to differences in efficacy.

Topical hemostatic agents may provide an economic advantage. Given the high cost associated with blood transfusion and its impact on hospital resources, the use of effective hemostats may be associated with potential cost savings due to reductions in transfusions [29, 30]. Although our study did not include a formal cost-utility and cost-efficacy analysis, we applied equal volumes (5 ml) in the two groups to increase comparability. In a tertiary-care hospital, CHM costs 40 USD per 1 ml, and GHM costs 80 USD per 1 ml. Thus, if CHM shows the same hemostatic effect as GHM, it may be more cost effective. Furthermore, collagen, one of the main ingredients of CHM, is one of the primary extracellular proteins in animal tissues, allowing for easy extraction and purification. Moreover, it provides an environment for fibroblast formation and induces wound healing by inactivating elastase and matrix metalloproteases.

The available topical hemostatic agents have demonstrated variable efficacy, may require significant preparation time, and provide limited benefit in diffuse, aggressive or difficult-to-access bleeding sites. However, CHM can easily and quickly access bleeding sites due to its flowable nature and short preparation time of less than 20 s. These strengths can be applied in the surgical field.

Our study includes some limitations. First, we had a small sample size. This fact may have led to a type II error, slightly narrowing the generalizability of the results found. Given the lack of clear differences between the two groups for ITT analysis, a larger number of patients is needed to evaluate the hemostatic superiority of both agents. Second, we did not conduct preoperative point of care testing for coagulopathy analysis. Third, this study had a short follow-up period; thus, the influence of the hemostats on the long-term clinical outcomes should be further evaluated. Lastly, we didn’t perform cost analysis in our trial, since this was not the focus of this research. It is known that CHM presents lower costs compared to GHM. We could say that in the future, it would be important to study our intervention with a bigger sample size and longer follow-up.

## Conclusion

This prospective, randomized, controlled trial indicates that CHM efficiently stops the bleeding of proximal anastomoses during CABG and may be useful for high-pressure anastomoses and multiple suture lines. In our study cohort, the time for successful hemostasis (mean 1.35 min) was comparable between CHM and GHM. Furthermore, CHM was associated with a decreased requirement for FFP and platelet transfusion. Thus, the hemostatic efficacy of CHM was found to be non-inferior to those of GHM in patients who had off-pump coronary grafting surgery.

## Data Availability

All data generated or analyzed during this study are included in this published article [and its supplementary information files].

## Abbreviations

ACT: Activated clotting time
aPTT: Activated partial thromboplastin time
CABG: Coronary artery bypass surgery
CHM: Collagen hemostatic matrix
FFP: Fresh frozen plasma
GHM: Gelatin hemostatic matrix
ICU: Intensive care unit
Hb: Hemoglobin
ICU: Intensive care unit
IRB: Institutional review board
RBC: Red blood cell
OPCAB: Off-pump coronary artery bypass
