# Correction to: Hemostatic efcacy of a fowable collagen-thrombin matrix during coronary artery bypass grafting: a double-blind randomized controlled trial

**DOI:** 10.1186/s13019-023-02360-9

**Published:** 2023-09-15

**Authors:** Hyo-Hyun Kim, Kang Ju Lee, Dae Ryong Kang, Jun Hyeok Lee, Young-Nam Youn

**Affiliations:** 1 Division of Cardiovascular Surgery, Severance Cardiovascular Hospital, Yonsei University College of Medicine, Yonsei University Health System, 250 Seongsanno, Seodaemun-gu, Seoul, 03722 Republic of Korea; 2 Department of Biostatistics, Wonju College of Medicine, Yonsei University, Wounju, Republic of Korea; 3 Department of Cardiothoracic Surgery, Ilsan Hospital, National Health Insurance Service, Goyang-si, 10444 Republic of Korea

**Correction to: Journal of Cardiothoracic Surgery (2023) 18:193** 10.1186/s13019-023-02196-3

Following publication of the original article [1], in this article text in Background section need to be changed from “Collastat®, Darim Tissen, Inc., Seoul, Korea; FDA premarket approval P810006S085” to “CollaStat, Dalim Tissen Co., Ltd., Seoul, Korea”. Also, there is a change in the Fig. 2 and the same has been placed below:

**Fig. 2:**
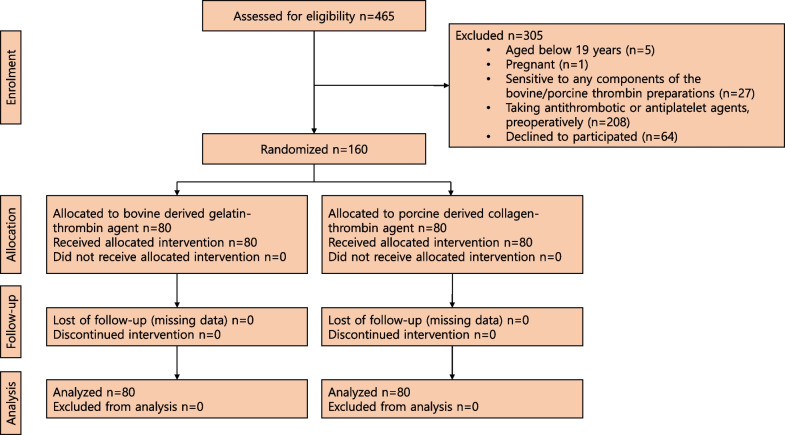
Study flow diagram. CAOD: coronary arterial obstructive disease, OPCAB: Of-pump coronary artery bypass

The original article has been corrected.

## References

[1] Kim HH, Lee KJ, Kang DR (2023). Hemostatic efficacy of a flowable collagen-thrombin matrix during coronary artery bypass grafting: a double-blind randomized controlled trial. J Cardiothorac Surg.

